# Fear of stigma from health professionals and family/neighbours and healthcare avoidance among PLHIV in Morocco: results from the Stigma Index survey Morocco

**DOI:** 10.1186/s12889-022-14010-1

**Published:** 2022-09-08

**Authors:** Rosemary M. Delabre, Amal Ben Moussa, Virginie Villes, Mohammed Elkhammas, Lahoucine Ouarsas, Daniela Castro Rojas Castro, Mehdi Karkouri

**Affiliations:** 1 Coalition PLUS, Community-Based Research Laboratory, Pantin, France; 2 Association de Lutte Contre le Sida, Casablanca, Morocco; 3 Aix Marseille Univ, Inserm, IRD, SESSTIM, Sciences Economiques & Sociales de la Santé & Traitement de L’Information Médicale, ISSPAM, Marseille, France

**Keywords:** HIV, Stigma, Key Populations, Healthcare access, Morocco

## Abstract

**Background:**

Enacted or anticipated stigma among people living with HIV (PLHIV) can negatively impact healthcare engagement. We identified factors associated with having avoided HIV health services for fear of stigma among PLHIV in Morocco.

**Methods:**

The Stigma Index survey was conducted in Morocco in March-June 2016. Factors associated with avoiding HIV testing and treatment services for fear of stigma by (A) health personnel or family/neighbours and (B) health personnel and family/neighbours compared to people who did not avoid health services for fear of stigma from either of the two sources were assessed using multinomial logistic regression models.

**Results:**

Among 583 respondents, 280 (48.0%) were women and median number of years living with HIV was 5[IQR:2–7]. Half of the respondents reported avoiding health services for fear of stigma by health personnel and/or family/neighbours: (A) *n* = 228, 39.1% and (B) *n* = 68, 11.7%. After adjustment on perceived health status, not having had easy access to antiretroviral treatment ((A) aRR [95% CI] = 1.76[1.16; 2.68]; (B) 2.18[1.11; 4.27]), discrimination by PLHIV ((A) 1.87[1.12; 3.13]; (B) 3.35[1.63; 6.88]) and exclusion from social activities ((A) 1.70[1.10; 2.61]; (B) 2.63[1.39; 5.00]) were associated with having avoided health services for fear of stigma by health personnel or/and family/neighbours. Being female (2.85[1.48; 5.47]), not having been referred for an HIV test for suspected symptoms 3.47[1.67; 7.22], having discussed sexual/reproductive health with a health professional (4.56[2.38; 8.71]), and not having the feeling to influence decisions on local projects for PLHIV (3.47[1.37; 7.83], were associated with having avoided health services for fear of stigma by both sources.

**Conclusion:**

Results suggest a cumulative effect of fear of stigma and discrimination among PLHIV in Morocco. PLHIV who have experienced discrimination may seek to avoid similar situations at the expense of their health. These results should inform multi-level interventions and broader advocacy efforts to reduce stigma and discrimination.

## Background

Although HIV prevalence in the Middle East and North Africa (MENA) region is relatively low (< 0.1%), recent data shows a 22% increase in new HIV infections over the last decade (2010–2019) [1]. New HIV infections are largely concentrated among key populations and their sexual partners: men who have sex with men, people who inject drugs, sex workers and their clients/sexual partners. Women, who represent one-third of new HIV infections in the region, are considered particularly vulnerable to HIV due to gender-based violence, in addition to other social, cultural and economic factors [2]. The stigma and discrimination experienced by key populations is a factor that increases vulnerability to HIV infection and impacts healthcare access in the MENA region, as in many other regions of the word [3]. Furthermore, people living with HIV (PLHIV) may experience intersectional or “layered stigma” due to their serostatus and for identifying, or being identified, with marginalised populations.

For decades, HIV has been shadowed by an “epidemic of fear, stigmatization and discrimination,” creating a major barrier for an effective and humane response that respects equality and human rights [4, 5]. Health-related stigma refers to “a social process or related personal experience characterized by exclusion, rejection, blame, or devaluation that results from experience or reasonable anticipation of an adverse social judgment about a person or group identified with a particular health problem” [6]. In the past few decades, several conceptual frameworks have been put forth to understand the pathways and underlying mechanisms of HIV-related stigma and its impact on the lives of PLHIV [7–10]. The negative impact of stigma on the health outcomes of PLHIV has been widely documented in the literature [11, 12]. Additionally, there is evidence that the source of stigma is an important factor to take into account. For example, stigma by healthcare workers has been shown to impact patient-provider relationships, as well as mental health and antiretroviral adherence among PLHIV [9, 13]. Social support from family/friends has also been identified as a key element for access and linkage to care among PLHIV [14]. Conversely, experience or fear of HIV-related stigma may lead to nondisclosure, and consequently, to poorer health outcomes due to lack of social support [15, 16]. Furthermore, there is evidence that a cumulation of stigma experiences may lead to poor engagement or disengagement from HIV care [14].

Stigma cannot be considered without taking into account the socio-cultural and institutional context in which it is experienced. A recent systematic review showed that despite some evidence of positive attitudes towards PLHIV and decreasing stigma and discrimination, there remains an overall negative attitude towards PLHIV in the MENA region [17]. The analysis also showed inadequate knowledge of HIV, which may be due to socio-cultural barriers to effectively communicate on taboo subjects such as premarital sex or homosexuality [17]. Socio-cultural factors may also impact HIV epidemiological data in the region, by which fears of stigma and discrimination and/or criminalisation for socially or culturally unacceptable HIV risk behaviors may lead to nondisclosure of true transmission routes [18, 19].

Although Morocco has shown considerable success in lowering the number of HIV infections in the last decade, new HIV infections persist among key populations and their sexual partners [20]. Additionally, key populations and PLHIV continue to experience stigma from family members, within the community and in the healthcare setting [21]. A recent study conducted in the country shows 29.9% of people who inject drugs, 23.4% of sex workers, and 7.9% of men who have sex with men reported avoiding healthcare services due to stigma and discrimination [20]. The lack of published data regarding HIV-related stigma in the region, in addition to the socio-cultural context, represent significant barriers to improving engagement in HIV care and treatment. In the present analysis, we aimed to evaluate the level and cumulative effect of healthcare avoidance for fear of stigma from family or neighbours and/or healthcare personnel among PLHIV in Morocco and to identify the factors associated. 

## Methods

Data was collected using the *People Living with HIV Stigma Index,* a tool developed through a partnership of several organizations (Global Network of People Living with HIV, International Community of Women living with HIV/AIDS, UNAIDS) which aims to collect evidence of HIV-related stigma among PLHIV. The PLHIV Stigma Index has been used all over the world to not only document HIV-related stigma but also to inform advocacy efforts to protect the human rights of PLHIV and to identify and develop strategies to address stigma and discrimination [22]. Implication of PLHIV, at all stages of the research process, is the core element in the Stigma Index survey.

The Stigma Index was implemented in Morocco between September and October 2016 following ethics approval from the ethics committee for biomedical research in Casablanca. The study aimed to document and evaluate the level of stigma and discrimination experienced by PLHIV in Morocco. Specific objectives included exploration of fear of stigma from health professionals and/or family/neighbours and health care avoidance. Although a qualitative study was also conducted within the context of this project, the present study focuses on the quantitative study data.

### Study preparation

The Association de Lutte Contre le Sida (ALCS), the first NGO operating in HIV/AIDS in Morocco and in the MENA region, and founding member of Coalition PLUS, worked in collaboration with the Ministry of Health, UNAIDS and the Global Fund, to conduct the study. Specific details regarding the preparatory phase of the study have been published previously [19]. Briefly, ALCS organized a preliminary workshop to develop a stigma training module and to discuss the necessary adaptations to the Stigma Index protocol to fit implementation in Morocco [19]. In line with the fundamental principle of the the People living with HIV Stigma Index (“for, by and with PLHIV” [23], 15 interviewers living with HIV and 5 supervisors were selected and trained by the ALCS to administer the study questionnaire.

### Sample size and site selection

Study participants had to meet the following inclusion criteria: PLHIV, aged 18 years or older, followed in a center that provides care for PLHIV (regardless of treatment) and provide consent to participate in the study. Participants were recruited in medical care centers across 8 cities in Morocco. A study sample size of 640 was determined across 9 sites (Casablanca, Agadir, Rabat, Marrakech (2 sites), Fez, Tangier, Nador and Beni Mellal) which were specifically selected to obtain a nationally representative sample of PLHIV. A randomly drawn cluster of 10 PLHIV among outpatients, consisting of 5 men and 5 women, was drawn at each participating medical care center on a given day. Further details regarding sampling can be found elsewhere [19]. A prior analysis has confirmed that the sociodemographic characteristics of the study sample did not significantly differ from the cohort of PLHIV on antiretrovirals in 2016.

### Measures

The People Living with HIV Stigma Index questionnaire was adapted and translated into French and Moroccan Arabic dialect "darija”. The adapted version was piloted among a dozen PLHIV in Casablanca.

Fear of stigma from health professionals and/or family/neighbours was evaluated in the questionnaire with the following questions: “Have you ever avoided seeking HIV testing/ prevention/treatment services out of fear or concern of stigma from health professionals ?” and “Have you ever avoided seeking HIV testing/prevention/treatment services out of fear or concern of stigma from family or neighbours ?”. We created the dependent variable in the following 3 classes: (1) no fear of stigma from neither health professionals nor family/neighbours; (2) fear of stigma from either health professionals or family/neighbours; and (3) fear of stigma from both health professionals and family/neighbours.

*The People Living with HIV Stigma Index* questionnaire collected information across several themes including: sociodemographic characteristics (gender, age, education, employment status, place of residence, household income in Moroccan dirham (MAD) per month), relationship status, sex work, number of years living with HIV, HIV test referral (for symptoms suspected to be HIV-related), access to antiretroviral treatment, discrimination by other PLHIV, social exclusion, general perceived health status, sexual and reproductive health discussions with a health professional and influence on decisions regarding local projects for PLHIV.

### Data analysis

Continuous variables were reported as median with IQR and categorical variables as frequencies. The three groups of “having avoided health services for fear of stigma” (as defined earlier) were compared using Chi-2 tests for categorical variables and Wilcoxon-Mann–Whitney tests for continuous variables. Factors associated with having avoided HIV testing and treatment services for fear of stigma were assessed using multinomial logistic regression models with RR (Risk Ratio/Relative Risk). We compared respondents who did not avoid health services for fear of stigma from either of the two sources of stigma (reference group) to those who avoided health services for fear of stigma from (A) health personnel or family/neighbours and (B) health personnel and family/neighbours (cumulative effect). Variables with a *p*-value lower than 0.25 in the univariable analysis were considered eligible to enter the multivariable model. In the multivariable analysis, we identified the main characteristics independently associated with (A) and (B), compared to those who did not avoid health services for fear of stigma from either of the two sources of stigma (reference). A backward procedure based on the Likelihood Ratio Chi-2 test was used to select variables for the final model. The final model selection was based on statistical significance (*p* < 0.05) and/or pertinence of the variables. A sensitivity analysis was conducted to take into account the cluster effect of site recrutement. Data analysis was carried out using Stata/SE 14.0 software (StataCorp LP, College Station, USA).

## Results

A total of 626 PLHIV were recruited to the study. For this analysis, 2 participants who identified as transgender were excluded due to the inability to analyze as a separate group. This analysis was based on 583 PLHIV who had complete data concerning the fear of stigma by health personnel and family/neighbours.

### Characteristics of participants

Among 583 participants, 280 (48.0%) were women and the median age was 36 [IQR 29–43] years (Table 1). One quarter (25.4%) had no formal education, close to half (49.5%) were unemployed, 71.3% lived in a big city or in a metropolis and median household income per month was 2000 MAD [1000—3000] (minimum wage was 2600 MAD at the time of the study). More than half (55.2%) of the respondents were single, divorced or separated. Sex work was reported by 14.1%. Median number of years living with HIV was 5 [IQR 2–7], 38.3% had been referred for a HIV test for symptoms suspected to be HIV-related and only 37.6% reported having easy access to antiretroviral treatment. 18.0% were discriminated against by PLHIV at least once. More than a quarter (27.6%) reported being excluded from social activities at least once. Almost half of the participants (49.1%) perceived their current health status to be excellent, very good or good. A third (34.4%) reported having discussed sexual and reproductive health with a health professional and 24.7% felt they had the power to influence decisions on local projects for PLHIV.

**Table 1:** Description and comparison of demographic, behavioural and social characteristics of the three groups of “having avoided health services for fear of stigma” (neither of the two, from health personnel or family/neighbours, and from health personnel and family/neighbours), among Stigma Index Morocco respondents, *N* = 583

	**Neither of the two (*n* = 287, 49.2%)**	**Health personnel or family/neighbours (*n* = 228, 39.1%)**	**Health personnel and family/neighbours (*n* = 68, 11.7%)**	
	**n (%) or median [IQR]**	**n (%) or median [IQR]**	**n (%) or median [IQR]**	*P*-value*
*Gender*				**< 0.001**
Male	173 (60.3)	110 (48.2)	20 (29.4)	
Female	114 (39.7)	118 (51.8)	48 (70.6)	
*Age*	35 [28–42]	36 [29–43]	37 [30–44]	0.438
*Highest level of formal education completed*				**0.038**
No formal education	57 (19.9)	67 (29.4)	24 (35.3)	
Primary school	74 (25.9)	53 (23.2)	16 (23.5)	
Secondary school or technical college or University	155 (54.2)	108 (47.4)	28 (41.2)	
*Current employment status*				**0.003**
Full time	24 (8.4)	18 (7.9)	14 (20.6)	
Part-time	131 (45.8)	83 (36.4)	24 (35.3)	
Unemployed and not working at all	131 (45.8)	127 (55.7)	30 (44.1)	
*Place of residence*				0.571
Rural area or small town or village	84 (29.6)	60 (26.4)	22 (32.4)	
Big city or metropolis	200 (70.4)	167 (73.6)	46 (67.6)	
*Household income in MAD per month*^a^	2000 [1000–3500]	1500 [1000–3000]	2000 [1500–3700]	**0.032**
*Current relationship status*				**0.001**
Married or cohabitating	107 (37.6)	68 (30.0)	31 (45.6)	
Single or in a relationship but not living together	118 (41.4)	82 (36.1)	12 (17.6))	
Divorced or separated	42 (14.7)	49 (21.6)	17 (25.0)	
Widow or widower	18 (6.3)	28 (12.3)	8 (11.8)	
*Sex worker*				**0.001**
No	257 (90.2)	191 (84.1)	50 (73.5)	
Yes	28 (9.8)	36 (15.9)	18 (26.5)	
*Number of years living with HIV*	4 [2–7]	5 [2–8]	5 [3–7]	0.547
*Referred for a HIV test for symptoms suspected to be HIV-related*				**0.003**
Yes	122 (42.7)	85 (38.1)	14 (20.6)	
No	164 (57.3)	138 (61.9)	54 (79.4)	
*Easy access to antiretroviral treatment*				**0.001**
Yes	124 (43.5)	74 (32.9)	19 (28.4)	
No	115 (40.4)	105 (46.7)	44 (65.7)	
Do not know	46 (16.1)	46 (20.4)	4 (6.0)	
*Discriminated against by PLHIV*^a^				**< 0.001**
Never	250 (87.7)	174 (79.5)	44 (65.7)	
At least once	35 (12.3)	45 (20.5)	23 (34.3)	
*Excluded from social activities*^a^				**< 0.001**
Never	229 (79.8)	156 (68.4)	37 (54.4)	
At least once	58 (20.2)	72 (31.6)	31 (45.6)	
*General self-perceived health status (current)*				**0.001**
Excellent or very good or good	156 (54.5)	100 (44.6)	28 (41.2)	
Fair or poor	130 (45.5)	124 (55.4)	40 (58.8)	
*Discussed sexual and reproductive health with a health professional*^a^				**< 0.001**
Yes	92 (32.4)	62 (27.6)	44 (65.7)	
No	192 (67.6)	163 (72.4)	23 (34.3)	
*Feeling to influence decisions on local projects for PLHIV*				**0.015**
Yes	81 (28.5)	54 (23.9)	8 (11.8)	
No	203 (71.5)	172 (76.1)	60 (88.2)	

^*^The three groups are compared using Chi-2 tests for categorical variables and Wilcoxon-Mann–Whitney tests for continuous variables

^a^ In the last 12 months

### Avoiding HIV health services for fear of stigma by health personnel and/or family/neighbours

Half of the respondents reported avoiding health services for fear of stigma by health personnel and/or family/neighbours: (A) health personnel or family/neighbours (*n* = 228, 39.1%) and (B) health personnel and family/neighbours (*n* = 68, 11.7%). Among those who avoided health services for fear of stigma by health personnel or family/neighbours (A), 143 (62.7%) avoided for fear of stigma by health personnel and 85 (37.3%) for fear of stigma by family/neighbours.

### Univariable analysis

In the univariable analysis, being female, having no formal education, being a sex worker, not having easy access to antiretroviral treatment, having been discriminated against by PLHIV, having been excluded from social activities, and having fair or poor general health status were associated with fear of stigma by health personnel and/or family/neighbours (Table 2).

**Table 2:** Factors associated with having avoided health services for fear of stigma from ((A) health personnel or family/neighbours; (B) health personnel and family/neighbours) using bivariable multinomial logistic regression (RR = Risk Ratio/Relative Risk, *N* = 583)

	One of two fears of stigma (A) vs neither of the two (Reference)		Both (B) vs neither of the two (Reference)	
	**RR [95%CI]**	***P*-value**	**RR [95%CI]**	***P*-value**
*Gender*				
Male	1.00		1.00	
Female	**1.63 [1.15;2.31]**	**0.007**	**3.64 [2.05;6.46]]**	**< 0.001**
*Age* (for one year increase)	1.01 [0.99;1.03]	0.372	1.01 [0.99;1.04]	0.321
*Highest level of formal education completed*				
No formal education	**1.69 [1.10;2.59]**	**0.017**	**2.33 [1.25;4.35]**	**0.008**
Primary school	1.03 [0.67;1.58]	0.900	1.20 [0.61;2.35]	0.601
Secondary school or technical college or University	1.00		1.00	
*Current employment status*				
Full time	0.77 [0.40;1.49]	0.445	**2.55 [1.18;5.50]**	**0.017**
Part-time	**0.65 [0.45;0.94]**	**0.023**	0.80 [0.44;1.44]	0.458
Unemployed and not working at all	1.00		1.00	
*Place of residence*				
Rural area or small town or village	1.00		1.00	
Big city or metropolis	1.17 [0.79;1.73]	0.432	0.88 [0.50;1.55]	0.654
*Household income in MAD per month* (for one thousand unit increase)^a^	0.95 [0.88;1.02]	0.133	1.01 [0.94;1.09]	0.728
*Current relationship status*				
Married or cohabitating	1.00		1.00	
Single or in a relationship but not living together	1.09 [0.72;1.66]	0.673	**0.35 [0.17;0.72]**	**0.004**
Divorced or separated	**1.84 [1.10;3.06]**	**0.020**	1.40 [0.70;2.79]	0.343
Widow or widower	**2.45 [1.26;4.76]**	**0.008**	1.53 [0.61;3.86]	0.364
*Sex worker*				
No	1.00		1.00	
Yes	**1.73 [1.02;2.93]**	**0.042**	**3.30 [1.70;6.43]**	**< 0.001**
*Number of years living with HIV* (for one unit increase)	1.02 [0.98;1.05]	0.400	1.01 [0.96;1.07]	0.614
*Referred for a HIV test for symptoms suspected to be HIV-related*				
Yes	1.00		1.00	
No	1.21 [0.84;1.73]	0.301	**2.87 [1.52;5.40]**	**0.001**
*Easy access to antiretroviral treatment*				
Yes	1.00		1.00	
No	**1.53 [1.03;2.26]**	**0.033**	**2.50 [1.38;4.53]**	**0.003**
Do not know	**1.68 [1.02;2.76]**	**0.043**	0.57 [0.18;1.76]	0.326
Discriminated against by PLHIV^*^				
Never	1.00		1.00	
At least once	**1.85 [1.14;2.99]**	**0.013**	**3.73 [2.02;6.91]**	**< 0.001**
Excluded from social activities^*^				
Never	1.00		1.00	
At least once	**1.82 [1.22;2.72]**	**0.003**	**3.31 [1.89;5.78]**	**< 0.001**
*General self-perceived health status (current)*				
Excellent or very good or good	1.00		1.00	
Fair or poor	**1.49 [1.05;2.11]**	**0.027**	**1.71 [1.00;2.93]**	**0.049**
Discussed sexual and reproductive health with a health professional^*^				
Yes	0.79 [0.54;1.17]	0.238	**3.99 [2.28;7.00]**	**< 0.001**
No	1.00		1.00	
*Feeling to influence decisions on local projects for PLHIV*				
Yes	1.00		1.00	
No	1.27 [0.85;1.90]	0.240	**2.99 [1.37;6.54]**	**0.006**

^*^ In the last 12 months

Being divorced, separated or a widow(er) were associated with avoiding HIV services for fear of stigma by health personnel or family/neighbours. Having (current) full time employment, not having been referred for the HIV test for symptoms suspected to be HIV-related, having discussed sexual and reproductive health with a health professional and not having the feeling to influence decisions on local projects for PLHIV were associated with having avoided health services for fear of the two sources of stigma. No significant association was detected between age, place of residence, household income per month and number of years living with HIV, and avoidance of health services for fear of stigma.

### Multivariable analysis

The final multivariable model included 555 respondents (Table 3). In the multivariable analysis, not having had easy access to antiretroviral treatment ((A) aRR [95% CI] = 1.76 [1.16;2.68]; (B) 2.18 [1.11;4.27]), having been discriminated against by PLHIV ((A) 1.87 [1.12;3.13]; (B) 3.35 [1.63;6.88]) and having been excluded from social activities ((A) = 1.70 [1.10;2.61]; (B) 2.63 [1.39;5.00]), were associated with having avoided health services for fear of stigma by health personnel and/or family/neighbours. Being female (2.85 [1.48;5.47]), not having been referred for the HIV test for symptoms suspected to be HIV-related (3.47 [1.67;7.22], having self-perceived  fair or poor health status (2.26 [1.20;4.28]), having discussed sexual and reproductive health with a health professional (4.56 [2.38;8.71]), and not having the feeling to influence decisions on local projects for PLHIV (3.27 [1.37;7.83]),) were associated with having avoided health services for fear of the two sources of stigma. The results of the sensitivity analysis did not significantly differ with the final model.

**Table 3:** Factors independently associated with having avoided health services for fear of stigma from ((A) health personnel or family/neighbours; (B) health personnel and family/neighbours) using multivariable multinomial logistic regression (RR = Risk Ratio/Relative Risk, *N* = 555)

	One of two fears of stigma (A) vs neither of the two (Reference)		Both (B) vs neither of the two (Reference)	
	**aRR [95%CI]**	***P*-value**	**aRR [95%CI]**	***P*-value**
*Gender*				
Male	1.00		1.00	
Female	1.44 [0.99;2.10]	0.060	**2.85 [1.48;5.47]**	**0.002**
*Referred for a HIV test for symptoms suspected to be HIV-related*				
Yes	1.00		1.00	
No	1.31 [0.89;1.93]	0.169	**3.47 [1.67;7.22]**	**0.001**
*Easy access to antiretroviral treatment*				
Yes	1.00		1.00	
No	**1.76 [1.16;2.68]**	**0.008**	**2.18 [1.11;4.27]**	**0.024**
Do not know	**1.75 [1.01;3.05]**	**0.047**	0.51 [0.13; 1.95]	0.326
*Discriminated against by other PLHIV*^a^				
Never	1.00		1.00	
At least once	**1.87 [1.12;3.13]**	**0.017**	**3.35 [1.63;6.88]**	**0.001**
*Excluded from social activities*^a^				
Never	1.00		1.00	
At least once	**1.70 [1.10;2.61]**	**0.016**	**2.63 [1.39 5.00]**	**0.003**
*General self-perceived health status (current)*				
Excellent or very good or good	1.00		1.00	
Fair or poor	1.44 [0.97;2.13]	0.067	**2.26 [1.20;4.28]**	**0.012**
*Discussed sexual and reproductive health with a health professional*^a^				
Yes	0.82 [0.54;1.25]	0.360	**4.56 [2.38;8.71]**	**< 0.001**
No	1.00		1.00	
*Feeling to influence decisions on local projects for PLHIV*				
Yes	1.00		1.00	
No	1.19 [0.76;1.85]	0.447	**3.27 [1.37;7.83]**	**0.008**

^a^ In the last 12 months

## Discussion

The results of the present study highlight the cumulative effect of fear or concern of stigma and discrimination within the social network/community and healthcare settings among PLHIV in Morocco. Among this representative sample of PLHIV who are engaged in health care [19], half of the respondents reported avoiding HIV testing/prevention/treatment services for fear or concern of stigma by health personnel and/or family/neighbours. PLHIV who have been discriminated against and who have been excluded in social contexts may seek to avoid further discrimination or exclusion by avoiding healthcare services. In such circumstances, healthcare avoidance may impact health outcomes of PLHIV. These results highlight the need for social and structural interventions to reduce the stigma and discrimination experienced by PLHIV, to improve their quality of life and to facilitate HIV testing, entry and retention in the HIV care cascade in Morocco.

The results of this study add to existing evidence of the cumulative effect of stigma [14, 15]. Studies have highlighted potential differences in the impact of HIV-related stigma based on the source of stigma [13, 14, 24]. High levels of anticipated HIV-related stigma by family/friends or within the community has been linked to lower serostatus disclosure, healthcare disengagement and self-imposed isolation to avoid stigma [9, 25, 26]. A recent study of 251 men and women living with HIV in the United States reported that 23% disengaged from care over the course of the 18 months. These participants reported significantly more experiences of family-related stigma [14]. Another US study has shown that women participants living with HIV experienced marginalisation not only from people around them but in the healthcare settings due to privacy violations and disrespect for patient autonomy [27]. Healthcare avoidance may be a consequence of enacted stigma within the healthcare setting, resulting in an increase in anticipated stigma and impacting patient-provider relationships [13, 24].

The impact of healthcare avoidance due to the experience or fear of stigma may be seen in poor health outcomes due to delay of diagnosis, start of treatment, and disengagement from care [9, 14, 28]. Negative and discriminatory attitudes against PLHIV remain a major barrier to improving the health and quality of life of PLHIV and to reducing HIV transmission in the region [17]. In this study, fear of stigma from both health personnel and family/neighbours was associated with not being referred for an HIV test for suspected HIV-related symptoms and not having easy access to antiretrovirals. Although the HIV care cascade in Morocco has significantly improved in the last decade, an important gap in testing remains despite efforts to diversify and expand the HIV testing offer; more than 22% of PLHIV are still unaware of their HIV status in Morocco [29]. Anticipated stigma, however, may act as a barrier to HIV testing uptake among key populations [30]. Since its approval in 2015 by the WHO [31], Morocco has adopted the “test and treat” strategy, and ARVs are provided for free to all PLHIV in Morocco regardless of their nationality. Although Morocco had reached the goal of 90% of PLHIV who know their status are on treatment [29], in this study, only 37.6% of participants reported having easy access to antiretroviral treatment. Fear of HIV-related stigma may explain poor access to ARVs in conjunction with other accessibility factors such as distance to healthcare structures and costs related to transportation and accommodation [32].

Additionally, avoidance of healthcare services may contribute to a greater vulnerability of PLHIV in Morocco. This vulnerability is visible in the socio-economic characteristics (for example, low level of education, especially among women, and the financial status) of the study participants which are representative of PLHIV in Morocco [33]. Such socio-economic vulnerabilities, in addition to other cultural and structural factors, may have led to an increased risk of HIV exposure before infection, and may continue to contribute to excess burden of disease and poor health outcomes among PLHIV. This phenomenon, known as syndemic theory, is particularly pertinent to the situation of women in the MENA region, who may also experience gender-based inequalities due to social and cultural norms [2]. Our results support earlier evidence of healthcare avoidance due to HIV-related stigma among women [2]. Existing data suggests that HIV-related stigma appears to more negatively impact women compared to men in this region [34]. One recent study among women at risk or living with HIV in the MENA region found that more than half (54%) stated that violence or fear of violence had impacted their ability to protect themselves from or manage their HIV and 66% reported experiencing violence in the healthcare setting [35].

Finally, social exclusion is an integral component of stigma and is rooted and reinforced by the lack of social and political power of marginalised populations [36]. Laws penalising socially “devalued” practices such as non-marital sex and same-sex relationships result in the criminialisation of marginalised populations most affected by HIV in Morocco [37]. Respondents who declared not having the feeling to influence decisions on local projects for PLHIV were more likely to report healthcare avoidance due to fear of stigma from health personnel and family/neighbours. This may be a manifestation of the effect of social and structural stigma experienced by PLHIV. Regarding social exclusion, 27.6% of the respondents reported having been excluded from social activities at least once in the previous 12 months. Furthermore, 18.0% of repondents reported discrimination by another PLHIV at least once in the previous 12 months. Similar results were found in the Stigma Index surveys conducted in Egypt 12.5% [38] and Pakistan 27% [39]. The survey did not allow for further exploration of the reasons for the reported discrimination, therefore it is difficult to confirm if the respondent was discriminated against due to their serological status and/or to other factors such as belonging (or being identified as belonging) to other marginalised groups, socially unaccepted behaviors or other illnesses. A newer version of the Stigma Index (Stigma Index 2.0) aims to explore in more detail the intersection between HIV-related and key population-related stigmas [22]. More studies are needed to explore these relationships and their impact on PLHIV for the development of targeted interventions.

### Strengths and limitations

This study, based on a representative sample of PLHIV engaged in care, contributes to the limited data regarding the experience of HIV-related stigma in the MENA region [19]. Additionally, this study contributes to the other data collected with the Stigma Index survey tool (https://www.stigmaindex.org/). The Stigma Index is a validated and widely used tool to measure stigma, allowing collection of standardised data on the experience and impact of stigma among PLHIV. In this study PLHIV were trained as interviewers and thus played an active part of study implementation and data collection. This study is limited by the cross-sectional nature of the data; analysis of the changes in stigma and discrimination over time is not possible with this dataset. Engagement in healthcare has been described as a dynamic process in which patients may cycle between periods of engagement and disengagement [14]. Longitudinal data regarding experience of and fear of stigma among PLHIV would further the understanding of its impact on healthcare engagement and inform appropriate interventions. Transgender people may experience gender-based discrimination which negatively impacts their access to healthcare [40]. However, due to the limited number of transgender participants in this study, we were unable to analyse healthcare avoidance among this group. Future editions of the Stigma Index should include a larger proportion of transgender individuals to evaluate healthcare avoidance and identify the need for specific interventions. Additionally, this study lacks more detailed information regarding the source of stigma, for example, if the respondent avoided seeking HIV testing/prevention/treatment services out of fear or concern of stigma from a specific type of healthcare professional. Finally, this study was limited to PLHIV engaged in care. Data collection regarding the experience of stigma among those who are not engaged in healthcare is needed to generalise these results.

## Conclusions

The results of this study suggest that the cumulation of fear or concern of stigma and discrimination within the social network/community and healthcare settings negatively impacts healthcare engagement among PLHIV in Morocco. There is also evidence that the experience of stigma and discrimination, for example by other PLHIV and having been excluded from social activities, was associated with healthcare avoidance. Documentation of the experience of stigma among PLHIV and its impact on healthcare uptake is scarce in the MENA region. It is therefore important that future studies collect longitudinal data on the experience and fear of stigma among PLHIV to better understand its impact and document changes over time. Capacity-building programs targeting key populations in the region may address certain aspects of social stigmatisation, however, other culturally-adapted interventions targeting factors at all socio-ecological levels (individual, interpersonal, community, organisational and public policy) are needed to further reduce stigma and discrimination and lead to social transformation [17, 41]. Such multi-level interventions could lead to crucial improvements in the health and well-being of PLHIV. Finally, these multi-level interventions should be led by PLHIV and key populations affected by HIV, in addition to other stakeholders, to take into consideration the broader context in which stigma and discrimination is driven and sustained [8, 41].

## Data Availability

The data that support the findings of this study are available from the corresponding author upon reasonable request.

## Abbreviations

aRR: Adjusted Risk Ratio/Relative Risk
ALCS: Association de Lutte Contre le Sida
CI: Confidence Interval
IQR: Inter-Quartile Range
MAD: Dirham Marocain
MENA: Middle East and North Africa
MSM: Men who have Sex with Men
PLHIV: People Living with HIV
